# Artificial Intelligence for Natural Products Drug Discovery in Neurodegenerative Therapies: A Review

**DOI:** 10.3390/biom16010129

**Published:** 2026-01-12

**Authors:** Francesco Fontanella, Tiziana D’Alessandro, Emanuele Nardone, Claudio De Stefano, Caterina Vicidomini, Giovanni N. Roviello

**Affiliations:** 1 Department of Electrical and Information Engineering “Maurizio Scarano”, University of Cassino and Southern Lazio, 03043 Cassino, Italy; 2 Institute of Biostructures and Bioimaging-Italian National Council for Research (IBB-CNR), Via De Amicis 95, 80145 Naples, Italy

**Keywords:** neurodegenerative diseases, artificial intelligence, NPs, neuroprotection, machine learning, drug discovery

## Abstract

This review examines the application of Artificial Intelligence (AI) in the discovery and optimisation of neuroprotective natural products (NPs) for neurodegenerative diseases (NDDs), emphasising the transition from general computational drug discovery to AI-specific approaches designed to address the chemical complexity and bioactivity profiles of natural compounds. The discussion encompasses relevant datasets, AI models, illustrative case studies, and emerging protein and biological targets that may serve as potential points of intervention for the prevention and treatment of NDDs. The review is organised to guide the reader from foundational knowledge to applied strategies; it begins by outlining the chemical and biological principles underlying neuroprotective NPs, then presents AI-driven computational frameworks for NP discovery, followed by a detailed examination of recent case studies in NDDs. Subsequent sections address the key challenges, opportunities, and future directions in the field, concluding with an evaluation of prospects for interdisciplinary collaboration across medicinal chemistry, neuroscience, and artificial intelligence.

## 1. Introduction

Neurodegenerative diseases (NDDs), including Alzheimer’s disease (AD), Parkinson’s disease (PD), amyotrophic lateral sclerosis (ALS), and Huntington’s disease (HD), represent an escalating global health concern, particularly in ageing populations [1]. Despite substantial advances in clarifying their underlying mechanisms, currently available therapies remain largely symptomatic and are unable to halt disease progression. This scenario highlights the urgent need for genuinely neuroprotective interventions that can act early in the neurodegenerative cascade. This growing clinical challenge highlights the need to explore natural products (NPs) as promising sources of neuroprotective compounds. Within this framework, NPs have long served as important reservoirs of structurally diverse and biologically active compounds. Recent research highlights their potential in neurodegenerative conditions, attributing beneficial effects to mechanisms such as antioxidant activity, modulation of neuroinflammation, mitochondrial protection, and inhibition of protein aggregation [2,3,4]. However, despite their biological potential, translating NPs into effective therapies remains challenging due to bioavailability and pharmacological complexity. Parallel to these developments, artificial intelligence (AI) and machine learning (ML) are rapidly transforming the field of drug discovery. In this context, artificial intelligence emerges as a powerful tool for navigating the chemical complexity of NPs and predicting their neuroprotective potential. The convergence between natural-product science and AI-driven computational strategies is emerging as a promising direction in the development of NDD therapeutics. Advances in ML and deep learning (DL) now enable the exploration of extensive NPs databases, the prediction of biological activities, and even the generation of NP-inspired molecules with optimised characteristics. A notable example is the use of a DL-based generative model to design small-molecule mimetics of NPs targeting the retinoid X receptor (RXR). Two of the generated compounds exhibited measurable in vitro activity, illustrating the potential of AI to translate NPs’ chemical complexity into synthetically accessible, biologically active leads [5,6,7]. By complementing traditional quantitative structure–activity relationship (QSAR), docking, and molecular dynamics approaches, AI-driven methods offer a new dimension in predicting activity and designing optimised compounds. AI technologies have recently become capable of inferring chemical structures directly from DNA sequences, supported by standardised biosynthetic pathway datasets stored in public repositories. This capability facilitates dereplication and supports linking molecules to their biosynthetic genes; yet, prioritising the vast chemical diversity of NPs for drug development continues to be a major challenge. Concurrently, AI is advancing computational drug design by elucidating the relationships between chemical structure and biological activity, as well as predicting molecular targets of NPs. Traditionally, this field has relied on QSAR modelling and structure-based methods such as docking and molecular dynamics. AI-driven approaches expand these methodologies, enabling the prediction of activity for untested molecules and uncovering patterns in protein dynamics. These advances have contributed to progress in de novo molecular design, drug repurposing, and optimisation of compound structures to improve therapeutic profiles. As a result, a growing convergence is emerging between omics-guided NP discovery and computational drug design. ML increasingly supports not only the prediction of NP structures but also the inference of their pharmacological properties, thus accelerating innovation at their interface. Building on this evolving landscape, our previous review provided a broad overview of computational strategies in NDD drug discovery but only marginally addressed the role of NPs and did not specifically examine AI-driven approaches for NP-based neuroprotection [8]. To fill this gap, the present review focuses on AI-enabled strategies for identifying neuroprotective NPs in the context of NDDs. This review distinguishes itself from recent publications in the last three to five years, such as Mullowney et al. (2023) [7], which broadly surveys AI in NP drug discovery via omics. In contrast, we provide an NDD-centric integration of AI frameworks tailored to NP complexity, including datasets (e.g., COCONUT, NPASS), models (e.g., GNNs, transformers), case studies, emerging targets (e.g., tau, α-synuclein), and future prospects like digital twins, bridging gaps in prior works for a guided foundational-to-applied approach. It first outlines the current chemical and biological landscape of neuroprotective NPs and their mechanistic relevance to neurodegeneration. It then examines state-of-the-art AI frameworks tailored to NP-oriented drug discovery, including representation learning, generative modelling, network pharmacology, and multi-omics integration. Furthermore, it identifies emerging proteins and biological targets with potential applications in the prevention and treatment of NDDs. Finally, the review discusses recent case studies on the optimisation of NPs for NDDs, offering a balanced assessment of progress, challenges, and future opportunities.

## 2. Overview of Major NDDS and Contemporary Treatment Approaches

### 2.1. Alzheimer’s Disease (AD)

**Figure 1:**
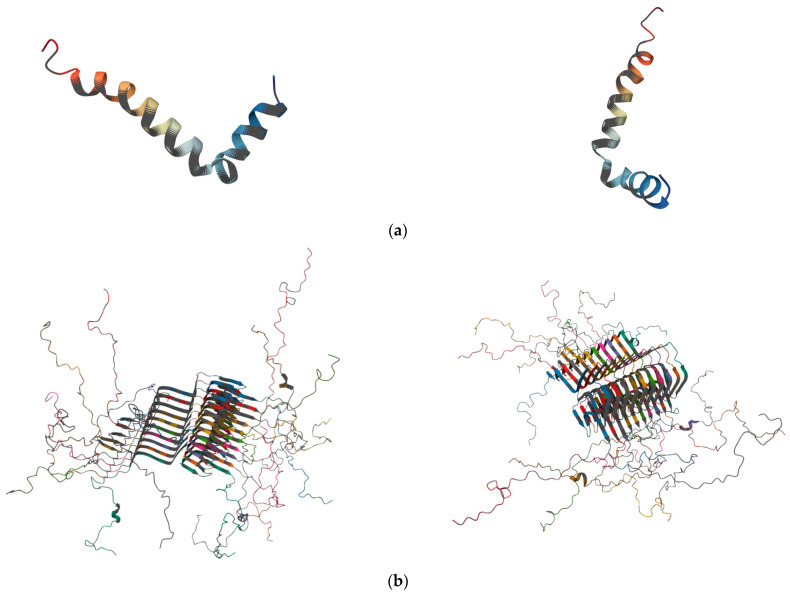
Three-dimensional structural representations of molecular biosystems central to neurodegenerative diseases: (**a**) amyloid-β peptide, associated with extracellular plaque formation in AD, and (**b**) α-Synuclein fibrils forming the core of Lewy bodies in PD and related synucleinopathies. The fibrillar conformations shown illustrate the β-sheet–rich architectures that promote aggregation, seeding, and neurotoxicity. Structures were obtained from the RCSB Protein Data Bank (https://www.rcsb.org/3d-view/1IYT/0 and https://www.rcsb.org/3d-view/2N0A/0, respectively, both accessed on 11 December 2025).

AD, first identified by Alois Alzheimer in 1907, is defined neuropathologically by the presence of extracellular plaques of amyloid-β (Aβ, Figure 1a) and intracellular neurofibrillary tangles [9,10].

Understanding the neuropathological hallmarks of AD is essential to unravel the cellular and molecular mechanisms underlying disease progression. According to the amyloid cascade hypothesis, Aβ accumulation triggers a sequence of events culminating in tau hyperphosphorylation, neuronal dysfunction, and cognitive decline. Nevertheless, considerable Aβ burden is often detectable in cognitively normal older adults, whereas tau pathology and neuronal loss correlate more closely with clinical symptoms [11]. These pathological features set the stage for a cascade of downstream events that drive neuronal dysfunction and cognitive decline. These observations have broadened AD pathophysiology to include downstream processes such as tau aggregation, chronic neuroinflammation, lipid dysregulation, and metal ion imbalance, while still recognising Aβ as an initiating component [9,10]. Beyond the core pathology, genetic and environmental factors modulate disease onset and severity. Familial AD due to mutations in *APP*, *PSEN1*, or *PSEN2* reinforces the role of Aβ overproduction in disease pathogenesis, although these cases account for less than 5% of total AD prevalence [12]. Familial and sporadic forms of AD illustrate how both inherited mutations and multifactorial influences converge on common pathways of neurodegeneration. In contrast, sporadic late-onset AD is multifactorial, shaped by ageing, genetics, environmental influences, and complex biological networks that regulate lipid metabolism, immune responses, synaptic maintenance, and endosomal–lysosomal trafficking [13]. Current therapeutic options remain primarily symptomatic [14]. Cholinesterase inhibitors and NMDA receptor antagonists offer modest clinical benefits, while recent anti-amyloid agents, such as lecanemab and donanemab, have renewed interest in Aβ-targeted strategies. A detailed understanding of these molecular pathways informs the development of targeted therapeutic strategies. However, optimal timing of intervention and long-term disease-modifying potential remain uncertain [15]. Tau pathology is increasingly recognised as a central amplifier of neurodegeneration, interacting with microglia and astrocytes to propagate inflammation and neuronal injury [16,17]. Impaired proteostasis, particularly dysfunction within the autophagy–lysosomal system, further limits the clearance of misfolded proteins. Meanwhile, genetic factors such as *APOE* ε4 influence lipid homeostasis, glial Aβ and tau clearance, and endosomal–lysosomal activity [18,19,20]. The AD drug development landscape reflects these expanding biological insights. The 2025 pipeline comprises 138 investigational agents across 182 clinical trials, with approximately 30% consisting of biological disease-targeted therapies and 43% consisting of small-molecule disease-targeted compounds. The remainder includes agents for cognitive enhancement (14%) and neuropsychiatric symptoms (11%). Despite the availability of symptomatic treatments, a pressing need remains for disease-modifying therapies, as reflected in ongoing clinical development efforts. Notably, about one-third of candidates are repurposed molecules, and 27% of ongoing studies employ biomarkers as primary endpoints, highlighting a shift toward precision-based approaches [21]. Current investigational agents build upon our growing knowledge of AD biology, integrating both small molecules and biologics aimed at multiple targets. Despite these advances, the likelihood of bringing multiple new disease-modifying therapies (DMTs) to market by 2025 remains limited, primarily because only compounds already in late Phase I or beyond are realistically positioned for regulatory evaluation within this timeframe. Efforts to accelerate AD therapeutic progress increasingly emphasise the need for improved trial design, enhanced clinical trial infrastructure, better-characterised participant registries, validated biomarkers for diagnosis and progression monitoring, and more sensitive clinical assessments. Addressing these challenges requires improved trial design, robust biomarkers, and collaborative research infrastructures. Broader scientific collaboration, greater standardisation, wider data sharing, and a deeper mechanistic understanding of disease onset and progression are essential to reducing development timelines and increasing success rates. Integrating mechanistic understanding with innovative clinical strategies may accelerate the development of effective interventions and reduce the societal impact of AD. Although substantial challenges remain, strengthening these research and clinical frameworks has the potential to lessen the societal burden of AD and support more effective therapeutic strategies in the years ahead.

### 2.2. Parkinson’s Disease (PD)

PD is characterised by the progressive degeneration of dopaminergic neurons in the substantia nigra pars compacta, leading to striatal dopamine depletion and the classical motor symptoms of bradykinesia, tremor, and rigidity. These neuronal alterations form the mechanistic basis of the observed clinical symptoms. Pathological hallmarks include intracellular α-synuclein (αSyn, Figure 1b) aggregates known as Lewy bodies, mitochondrial dysfunction, oxidative stress, impaired autophagy, and neuroinflammation [22,23,24,25,26]. These processes converge on key mechanisms of neuronal damage and progressive functional loss. Aberrant αSyn aggregation is also central to other synucleinopathies, including multiple system atrophy (MSA) and dementia with Lewy bodies (DLB). Since motor symptoms appear years after αSyn pathology begins and there is clinical overlap, early and accurate diagnosis remains challenging [27]. Timely detection of αSyn pathology is therefore crucial for effective therapeutic intervention. Meta-analytic data report sensitivities of ~86% and specificities of ~92% [27,28]. Biochemical and imaging biomarkers of dopaminergic dysfunction, including cerebrospinal fluid DOPA decarboxylase (DDC) levels, DAT PET/SPECT, and VMAT2 imaging, enable biologically based patient stratification and staging, thereby improving clinical trial design and sensitivity to disease-modifying interventions [29,30,31,32]. The integration of clinical and molecular biomarkers provides a solid foundation for more targeted and personalised therapeutic approaches. Current treatments, such as levodopa and dopamine agonists, offer symptomatic benefits but do not alter neurodegeneration or effectively address non-motor symptoms [33]. Consequently, there remains an urgent need to develop strategies that can slow or halt disease progression, building on a detailed knowledge of the underlying molecular mechanisms. These insights also pave the way for exploring neuroprotective NPs and AI-driven drug discovery as complementary avenues for innovative PD therapies.

### 2.3. Multiple System Atrophy

MSA is a rare, rapidly progressive synucleinopathy that typically manifests in adulthood and is characterised by a variable combination of Parkinsonism, cerebellar ataxia, and autonomic dysfunction. The parkinsonian variant (MSA-P) is characterised primarily by bradykinesia, rigidity, and postural instability, reflecting predominant striatonigral degeneration. In contrast, the cerebellar variant (MSA-C) presents mainly with gait and limb ataxia, dysarthria, and oculomotor abnormalities, consistent with olivopontocerebellar involvement. Although these subtypes differ in clinical presentation, both exhibit overlapping autonomic dysfunction. Pathologically, MSA is defined by the presence of cytoplasmic inclusions in oligodendrocytes, primarily composed of misfolded alpha-synuclein [34]. The distribution of α-synuclein pathology partially aligns with these phenotypes, but disease progression, prognosis, and response to symptomatic therapies remain similarly limited across both forms. According to the latest Movement Disorder Society diagnostic criteria, a diagnosis of clinically established MSA requires the presence of autonomic failure in conjunction with poorly levodopa-responsive Parkinsonism and/or cerebellar features [35]. While current symptomatic treatments can improve patients’ quality of life, their effects are often temporary and do not alter the underlying disease course. Therefore, there is an urgent need for therapeutic strategies that target the core pathogenic mechanisms underlying these conditions. Ongoing preclinical and clinical research is focusing on key aspects of MSA pathology, including abnormal protein aggregation, synaptic dysfunction, impaired proteostasis, neuroinflammation, and neuronal loss, with the goal of slowing or halting disease progression. Concurrently, efforts are being made to identify more specific and sensitive biomarkers for early diagnosis and disease monitoring [36]. Importantly, a growing number of novel disease-modifying candidates are under investigation, offering the potential for multi-targeted and personalised therapeutic approaches, despite the limited success of earlier interventions [37].

### 2.4. Amyotrophic Lateral Sclerosis (ALS)

ALS is a progressive neurodegenerative disease affecting upper and lower motor neurons in the brain, brainstem, and spinal cord, clinically manifesting as progressive muscle weakness and atrophy, often leading to respiratory failure within 3–5 years [38,39]. Approximately 10% of cases are familial (fALS), with the remainder being sporadic (sALS). Over 50 ALS-related genes have been identified, with the most frequent being *SOD1*, *TARDBP*, *FUS*, and [40]. Proposed mechanisms include the “dying forward,” “dying back,” and independent degeneration hypotheses [41,42]. Riluzole, a glutamate neurotransmission inhibitor, remains the only FDA-approved drug, offering modest survival benefits [43]. The antioxidant edaravone has also been approved, showing efficacy in slowing disease progression, particularly in early-stage ALS [43].

### 2.5. Huntington’s Disease (HD)

HD is a progressive, autosomal-dominant neurological disorder characterised by motor impairments, cognitive decline, and psychiatric manifestations [44]. The condition arises from an abnormal expansion of CAG repeats in exon 1 of the *HTT* gene, leading to the accumulation of mutant huntingtin (mHTT) fragments that cause neurotoxicity, primarily affecting cortical and striatal neurons. Current research has focused on developing strategies to reduce mHTT levels and mitigate disease progression, including small-molecule therapies, gene-based interventions, and protein degradation techniques. Emerging approaches, such as *CRISPR*-mediated gene editing and targeted small-molecule protein clearance, are also being investigated, with experimental compounds like VTX-003 and ANX005 showing promise in preclinical studies [45,46]. Despite these advances, challenges remain in terms of long-term efficacy, delivery methods, and minimising off-target effects. Overall, these findings highlight the continued need for therapeutic strategies that maximise safety and effectiveness, ultimately aiming to preserve neurological function and improve the quality of life for patients with HD [47].

## 3. Neuroprotective NPs: Classes, Sources, and Mechanisms

Natural products include structurally diverse molecules, such as flavonoids, polyphenols, alkaloids, triterpenoids, and phenolic derivatives, that show broad neuroprotective effects in NDDs, including PD, AD, HD, and other protein-related pathologies (Table 1). Despite their chemical heterogeneity, these compounds often converge on shared mechanisms that are central to neuroprotection. In particular, these compounds share common pathways, including antioxidant activity, anti-inflammatory effects, protection of mitochondrial function, modulation of autophagy, and interference with toxic protein aggregation. At the same time, each class displays distinctive molecular targets, offering complementary therapeutic potential. The following sections examine the significant classes of neuroprotective natural products, highlighting both overlapping and unique mechanisms that inform their potential therapeutic applications.

### 3.1. Flavonoids: Tangeretin, Nobiletin, Quercetin, and Luteolin

**Figure 2:**
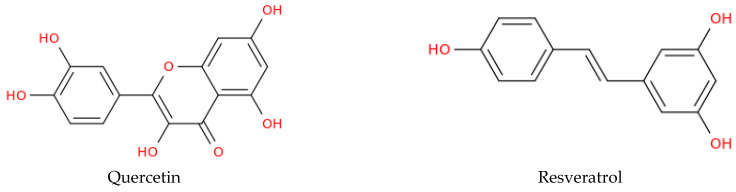
Chemical structures of quercetin and resveratrol, two dietary polyphenols widely investigated for their neuroprotective effects in neurodegenerative disorders. Both molecules share a polyphenolic scaffold with multiple hydroxyl groups, which underlies their antioxidant, anti-inflammatory, and mitochondrial-protective properties, as well as their ability to modulate protein aggregation and autophagy-related pathways.

Citrus flavonoids, such as tangeretin and nobiletin, have attracted significant attention due to their antioxidant and anti-inflammatory effects, as well as their ability to modulate pathways directly involved in the pathogenesis of AD and PD, including NMDA receptor signalling, amyloid-β deposition, tau hyperphosphorylation, and neprilysin-mediated amyloid clearance [48]. In PD models, tangeretin reduces inflammatory cytokines such as IL-1β, IL-6, and IL-2 and prevents dopaminergic neuron loss in MPTP-treated rodents [49]. Nobiletin similarly exhibits robust neuroprotection; in MPP^+^-treated rats, a 10 mg/kg dose preserves substantia nigra dopaminergic neurons, suppresses microglial activation, and maintains GDNF levels [118]. In MPTP-treated mice, nobiletin promotes tyrosine hydroxylase phosphorylation via CaMKII and PKA, stimulates dopamine release through voltage-dependent calcium channels, and ameliorates motor and cognitive deficits [119]. Both tangeretin and nobiletin attenuate Aβ_1–42_-induced neurotoxicity; tangeretin primarily inhibits Aβ aggregation, whereas nobiletin reduces oxidative stress [50]. Quercetin (Figure 2) shares several mechanistic features with citrus flavonoids, particularly in terms of antioxidant, anti-inflammatory, and mitochondrial-protective actions, but exhibits unique effects on mitophagy and neurotrophic signalling.

In AD models, quercetin reduces Aβ aggregation, decreases oxidative stress, and enhances memory consolidation and synaptic plasticity [59]. Luteolin, a dietary flavone with high bioavailability, crosses the blood–brain barrier (BBB) and exhibits neuroprotective, anti-inflammatory, and antioxidant properties [60]. In cellular models, it decreases inflammatory-mediated rotenone toxicity, reduces LDH release, and increases Nrf2 and Trx1, preserving redox balance [61]. In vivo, luteolin ameliorates cognitive deficits in chronic cerebral hypoperfusion, normalises glial activation, reduces inflammatory cytokines, and boosts SOD and GPx activities [120]. It prevents 6-OHDA-induced apoptosis by modulating Bax, Bcl-2, and p53, and preserves dopaminergic neurons [121]. Luteolin also reduces amyloid-β aggregation and neuroinflammation in AD models and mitigates huntingtin aggregation and motor deficits in HD models [63,64]. Together, these flavonoids exemplify how multiple compounds can act on overlapping pathways while also engaging unique mechanisms, providing a foundation for combinatorial or synergistic therapeutic strategies.

### 3.2. Polyphenols: Resveratrol, Curcumin, Caffeic Acid, CAPE, Ferulic Acid, EGCG

In addition to flavonoids, polyphenols represent a widely studied group that shares many neuroprotective mechanisms while acting on metabolic and autophagy-related pathways characteristic of this class. Resveratrol and quercetin (Figure 2), two of the most extensively investigated dietary polyphenols, modulate multiple pathological mechanisms underlying protein aggregation, mitochondrial dysfunction, and chronic neuroinflammation [122]. Resveratrol regulates oxidative stress, apoptosis, and mitochondrial turnover; enhances antioxidant defences; stimulates mitophagy via SIRT1 and AMPK/ERK signalling; and facilitates α-synuclein clearance through autophagy [65]. In rotenone and 6-OHDA PD models, resveratrol preserves dopaminergic neurons, restores striatal dopamine, and improves motor and cognitive performance [65,66,123]. Resveratrol also downregulates pro-inflammatory mediators such as COX-2 and TNF-α, exerts epigenetic effects, and demonstrates enhanced gut–brain axis interactions and bioavailability when delivered through cyclodextrin-based carriers [67,68,69]. In AD models, resveratrol reduces the amyloidogenic processing of APP, promotes amyloid-β clearance, and limits the propensity for aggregation. It supports neuronal health through antioxidant effects, improves cognition, and enhances antioxidant enzyme activity via SIRT1-dependent autophagy and mitochondrial support [70,71,72]. Beyond classical polyphenols, additional phenolic subclasses further diversify these neuroprotective profiles while maintaining key mechanistic intersections. These polyphenols highlight how natural products can simultaneously modulate multiple disease-relevant pathways, reinforcing their therapeutic promise across NDDs. Other natural product classes, including traditional plant extracts, terpenoids, and glycosides, share core antioxidant, anti-inflammatory, and anti-apoptotic effects, though each displays unique target specificities and experimental contexts. Additional polyphenols such as salvianolic acids, caffeic acid (CA), caffeic acid phenethyl ester (CAPE), ferulic acid, curcumin, EGCG, luteolin, and oleuropein converge on similar neuroprotective mechanisms across PD and AD models. CA exhibits antioxidant, anti-inflammatory, and anti-amyloid effects, preserves mitochondria and synapses, and improves cognition [77,78,79,80]. CAPE enhances Nrf2/HO-1, reduces apoptosis and inflammation, and rescues memory deficits [81,82]. Curcumin inhibits Aβ and tau aggregation, counteracts oxidative stress, and modulates neuroinflammatory pathways, although its effects are limited by poor bioavailability [73,74]. To address such limitations, advanced AI frameworks, such as evolved transformer models, are now being utilized to more accurately predict ADME/Tox constraints early in the drug development process [124]. Collectively, these examples illustrate the capacity of natural products to address multiple aspects of neurodegeneration, supporting their inclusion in multi-target therapeutic strategies.

### 3.3. Olive-Derived Phenolics: Oleuropein and Derivatives

Olive-derived phenolic compounds represent a subgroup combining the antioxidant and anti-inflammatory activities of polyphenols with additional effects on mitochondrial dynamics and protein aggregation. Oleuropein and its derivatives, phenolic compounds from olive leaves and oil, cross the BBB and modulate oxidative stress, inflammation, and apoptosis. In rotenone PD models, oleuropein improves motor performance, upregulates CREB, BDNF, TH, phosphorylated GSK-3β, TrkB, and Akt, reduces α-synuclein, Bax, and caspase-3, and increases Bcl-2 [86]. In *C. elegans*, oleuropein aglycone and hydroxytyrosol extend lifespan, reduce α-synuclein aggregation, and protect dopaminergic neurons [87]. In vitro, they limit ROS production, prevent DNA damage, and modulate Drp1-dependent mitochondrial dynamics [88,89,90]. In AD models, oleuropein interferes with amyloid-β oligomerisation, reduces oxidative stress, and protects neurons [91]. Parallel to olive phenolics, ginger-derived phytochemicals offer a complementary yet mechanistically aligned neuroprotective profile, particularly emphasising microglial and inflammatory modulation. Together with other phenolics, olive-derived compounds exemplify how natural products can integrate multiple protective mechanisms into a single molecule.

### 3.4. Ginger-Derived Compounds: 6-Gingerol, 6-Shogaol, Zingerone

Ginger-derived compounds—including 6-gingerol, 6-shogaol, and zingerone—protect neurons by reducing ROS, inflammation, and microglial hyperactivation while enhancing dopamine release [98]. 6-Gingerol improves cognition, reduces IL-6, TNF-α, and iNOS, and promotes neuronal survival by suppressing SAPK/JNK and increasing survivin [92,93,94]. 6-Shogaol inhibits iNOS, NO, COX-2, p38 MAPK, NF-κB, and PGE_2_, decreasing TNF-α and IL-1β in vitro and in vivo [95,96]. Together with these plant-derived phenolics, additional natural product classes contribute further mechanistic breadth, often integrating antioxidant and anti-inflammatory actions with distinct pathway-specific effects. These findings illustrate how dietary phytochemicals can modulate inflammation and oxidative stress, complementing other neuroprotective strategies.

### 3.5. Ginkgo Biloba, Ursolic Acid, Ginsenosides

Ginkgo biloba leaves (GBLs) modulate oxidative stress, neuroinflammation, neurotransmission, and platelet-activating factor (PAF), improving cognition, behaviour, and neuropsychiatric symptoms in preclinical and clinical NDD studies [101]. Ursolic acid and its derivatives, as well as ginsenosides from ginseng, act through anti-apoptotic, antioxidant, and anti-inflammatory pathways to support neuronal homeostasis and cognitive function [104,107]. Building on these terpenoid- and glycoside-based neuroprotective effects, the next class, alkaloids, adds another mechanistic dimension, particularly through cholinesterase modulation. This class highlights the therapeutic potential of terpenoid- and glycoside-based compounds in multi-target neuroprotection.

### 3.6. Alkaloids: Berberine, Huperzine A, Harmine, and Others

Alkaloids, including berberine, huperzine A, harmine, and others, inhibit acetylcholinesterase and butyrylcholinesterase, restore cholinergic function, and protect neurons via antioxidant and anti-inflammatory mechanisms [110,111,125]. As such, alkaloids add another mechanistic layer, highlighting the capacity of NPs to target distinct yet complementary pathways relevant to neurodegenerative disease progression. Taken together, these classes of natural products illustrate how structurally diverse molecules can converge on a core set of neuroprotective mechanisms while preserving distinct pathway selectivities. This integrated perspective highlights the promise of natural products as complementary multi-target therapies capable of addressing the complex pathogenesis of neurodegenerative diseases. Many NPs are described as ‘multi-target’ compounds because they are capable of modulating several molecular pathways simultaneously. In many cases, this property is first inferred computationally through molecular docking, network pharmacology, and pathway-based analyses, which predict potential protein–ligand interactions and system-level effects. However, practical verification requires experimental confirmation, typically through biochemical assays assessing binding and enzymatic inhibition, phenotypic screening in cellular or organismal models, and multi-omics approaches that demonstrate convergent modulation of multiple biological pathways. Several NPs, including, for example, curcumin, have been experimentally shown to exert multi-target effects across different signalling cascades in both preclinical studies and clinical investigations [126,127].

## 4. AI-Driven Frameworks for Natural Product-Based Neuroprotective Discovery

The integration of artificial intelligence into NP research represents a fundamental shift from empirical screening toward data-driven discovery strategies for neuroprotective compounds [7]. NPs, derived from plants, marine organisms, fungi, and microbial sources, offer remarkable structural complexity and multi-target activities that make them particularly valuable for addressing NDDs [128]. However, their chemical diversity, limited availability, and complex mechanisms of action present substantial challenges for conventional drug discovery approaches [129,130]. Recent advances in artificial intelligence have created unprecedented opportunities to overcome these limitations through specialised datasets, sophisticated machine learning models, generative algorithms, and systems-level analytical frameworks [131,132]. These computational tools enable researchers to navigate the vast chemical space of NPs more efficiently, predict biological activities with greater accuracy, and design optimised analogues that retain beneficial properties while improving drug-like characteristics. The synergy between machine learning and natural products cheminformatics has proven particularly effective in lead compound discovery, where AI-driven approaches leverage the structural complexity and uniqueness of natural products to identify selectively binding compounds in the nanomolar range [133]. Machine learning models trained on diverse bioactivity datasets have demonstrated their capacity to model complex natural product structures and generalise findings to novel chemical and sequence spaces [134]. Furthermore, the integration of explainable AI methodologies allows researchers to identify characteristic structural motifs and molecular features that differentiate effective from ineffective compounds [135], facilitating both target identification and compound optimisation for neuroprotective applications. The following subsections examine the key components of this technological transformation, supported by recent advances and practical applications relevant to major NDDs, including AD, PD, and ALS.

### 4.1. AI Datasets and Representation Learning for NP Chemistry

The foundation of artificial intelligence applications in neuroprotective NP discovery rests upon datasets that capture both chemical structures and biological activities. These repositories provide the essential training data for machine learning models while enabling representation learning approaches that translate molecular structures into formats suitable for computational analysis. NPs present unique challenges for data representation due to their stereochemical complexity, diverse functional groups, and scaffolds that differ substantially from synthetic compounds [136]. Recent reviews have highlighted the potential of combining such chemical and biological data with AI-driven analytics to accelerate neuroprotective drug discovery. In particular, the authors of [137] critically review the neuroprotective effects of NPs derived from plants, marine organisms, and fungi in NDDs, emphasising mechanisms such as antioxidant, anti-inflammatory, and mitochondrial protective effects, while noting that challenges like bioavailability and the need for clinical validation can be addressed through AI-based modelling. In [7], it is further described how advancements in computational omics and AI technologies unlock hidden NP diversity and enable data-driven exploration for drug discovery, and [138] discusses how AI-driven analytics accelerate therapeutic development for NDDs. The Collection of Open NPs (COCONUT) database is the most extensive aggregated repository, containing over 400,000 unique NP structures compiled from multiple open-source databases. This resource offers critical annotations, including molecular properties, Murcko scaffold analysis, and natural product-likeness scores, which help distinguish these compounds from synthetic molecules. COCONUT supports artificial intelligence applications through deepSMILES (deep Simplified Molecular Input Line Entry System) representations compatible with DL architectures and ClassyFire chemical classifications that enable systematic organisation of structural features [136]. In the context of neuroprotective drug discovery, researchers have employed COCONUT to generate fragment libraries for virtual screening against protein targets implicated in neurodegenerative pathways, particularly those involved in amyloid aggregation characteristic of AD. Complementing structural databases, the NP Activity and Species Source (NPASS) database links over 35,000 NPs with quantitative bioactivity data against more than 5000 molecular targets. This resource uniquely combines chemical structures with biological source information and experimental activity measurements, providing essential training data for predictive models [139]. For neuroprotective applications, NPASS has proven particularly valuable in identifying compounds with antioxidant properties, including marine NPs showing promise for PD treatment through mitochondrial protection mechanisms [140,141]. The LOTUS Initiative represents a more recent advance in NP data integration, establishing a Wikidata-linked knowledge graph containing over 750,000 structure-organism-reference triplets. This resource employs standardised SMILES, which use brackets and parentheses to denote branches and ring closures and InChI notations for chemical structures, while incorporating NPClassifier, a deep neural network specifically designed for NP chemical classification [142]. The integration with Wikidata enables artificial intelligence-driven chemotaxonomy studies that connect neuroprotective scaffolds to their biological sources, facilitating biodiversity-guided discovery strategies that target organisms from specific ecological niches known to produce bioactive metabolites. Specialised databases focusing on microbial NPs provide additional resources for neuroprotective discovery. The NP Atlas catalogues over 30,000 microbial metabolites along with their associated biosynthetic gene clusters, enabling genome mining approaches that predict chemical structures directly from genetic sequences [143]. The Global NPs Social (GNPS) network improves these structural databases through mass spectral data integration, providing experimental validation for structure elucidation of novel neuroprotective compounds [144]. The NP Magnetic Resonance Database (NP-MRD) provides further support for structure confirmation through NMR spectral data, addressing the critical need for accurate structure determination in NP research. Representation learning techniques transform these diverse chemical structures into mathematical representations suitable for machine learning applications. Extended connectivity fingerprints capture local structural environments around each atom [145], while graph-based representations preserve the full molecular topology [146,147], including stereochemistry. Autoencoders and other DL architectures learn compressed representations that capture essential chemical features while reducing dimensionality [148,149]. These methods have proven particularly effective for NPs, successfully encoding complex features such as macrocyclic structures, unusual functional groups, and specific stereochemical configurations that influence BBB penetration, a critical requirement for neuroprotective therapeutics. Molecular weight represents a crucial differentiating attribute between drug-like and non-drug-like natural products, particularly for CNS applications. While most currently successful AI applications focus on NPs with MW < 600 Da (flavonoids, polyphenols, simple terpenoids, alkaloids), higher-MW natural products (peptides, macrocycles, glycosylated scaffolds; MW 700–1500 Da) frequently exhibit superior target affinity and selectivity but suffer severely restricted BBB penetration. Current representation learning methods (ECFP, graph neural networks, learned autoencoder embeddings) implicitly capture molecular size through atom/count-based features and graph diameter, yet rarely explicitly encode MW as a conditioned or weighted descriptor. This leads to systematic over-optimism in predicted BBB permeability and drug-likeness scores for medium-to-high MW scaffolds, limiting the effective chemical space explored by most published AI models in neuroprotective discovery. Despite these advances, significant challenges remain in NP data curation. Approximately 31 percent of entries in COCONUT lack complete taxonomic annotations, limiting biodiversity-guided discovery approaches [124]. Despite the increasing size of NP datasets, their impact on downstream experimental validation remains limited. Data quality issues, including structure errors, activity measurement variability, and incomplete metadata, continue to affect model performance. Across publicly reported AI-driven NP screening studies, only a minority of computationally prioritised compounds progress to experimental testing, largely due to dataset bias toward well-characterised, low-molecular-weight scaffolds and the scarcity of negative or failed examples. This bias inflates apparent model performance during internal validation while reducing real-world hit rates, particularly for structurally complex or underrepresented natural products. Recent developments have focused on addressing these limitations through community curation efforts, artificial intelligence-assisted literature mining, and the integration of multi-omics data, which connects chemical structures with biological pathways relevant to neurodegeneration [150].

### 4.2. Machine Learning for NP Bioactivity and Target Prediction

Machine learning models have transformed the prediction of NP bioactivities and molecular targets, providing essential tools for identifying neuroprotective candidates that modulate key pathological mechanisms in NDDs. These computational approaches address fundamental challenges in NP research, including the need to predict activities against multiple targets simultaneously, understand complex structure-activity relationships, and prioritise compounds for experimental validation from vast chemical libraries [151,152]. Graph neural networks (GNNs) have emerged as particularly powerful tools for NP bioactivity prediction, representing molecules as mathematical graphs where atoms form nodes and chemical bonds create edges [153]. This representation preserves the complete molecular topology while enabling learned features that capture complex structural patterns. The study proposed in [154] introduces a novel chain-aware GNN that improves molecular property prediction by specifically capturing chain structures and long-range dependencies within molecular graphs, addressing the limitations of conventional GNNs like feature squashing. The RetroGNN model exemplifies this approach [152], evaluating the synthetic accessibility of neuroprotective NP analogues by learning retrosynthetic patterns from reaction databases. GraphDTA extends these capabilities to drug-target interaction prediction, achieving superior performance for targets relevant to NDDs, including acetylcholinesterase, beta-secretase, and tau aggregation inhibitors. In a notable application, GNNs identified sclareol, a labdane diterpene from Salvia species, as a selective Cav1.3 calcium channel antagonist with potential therapeutic value for PD motor symptoms [155]. Transformer architectures, originally developed for natural language processing, have been successfully adapted to handle SMILES representations of chemical structures. These models treat molecular strings as sequences, learning context-dependent representations that capture long-range structural relationships. The MS2Mol framework demonstrates this approach by deriving NP structures directly from mass spectrometry data, supporting the discovery of neuroprotective peptides from marine organisms [156]. The Natural Product-Inspired Molecular Optimisation (NIMO) framework employs transformers to generate multi-target ligands specifically designed for AD, simultaneously optimising activity against amyloid-beta aggregation, tau phosphorylation, and neuroinflammatory pathways [157]. Classical machine learning approaches, including support vector machines and random forests, continue to provide value for quantitative structure-activity relationship modelling, particularly when training data are limited. These methods have successfully predicted anti-inflammatory activities of flavonoids and other polyphenolic NPs relevant to AD pathology [158]. The PASS (Prediction of Activity Spectra for Substances) system employs Bayesian approaches to predict biological activity profiles across thousands of activity types, enabling rapid screening of NP libraries for neuroprotective properties [159]. The STarFish platform extends these capabilities through ensemble learning methods that integrate multiple prediction algorithms, improving reliability for novel NP scaffolds. Integration of genomic, transcriptomic, and metabolomic data through multi-view learning approaches further enhances prediction accuracy by capturing the relationship between biosynthetic capacity and actual metabolite production under specific conditions [160]. Hybrid models combining GNNs with transformer architectures represent the latest evolution in NP bioactivity prediction (Table 2). These approaches use the complementary strengths of both architectures—graphs for capturing molecular topology and transformers for learning sequential patterns in SMILES representations [161]. Such models have demonstrated improved accuracy in predicting BBB penetration, a critical property for neuroprotective drugs, by learning from datasets that combine chemical structures with experimental permeability measurements. In [162], the authors demonstrate that a Stacking Ensemble machine learning model, utilising a large dataset of bioactive compounds, significantly outperforms GNNs, Transformers, and traditional methods in predicting pharmacokinetic parameters with high accuracy, thereby showing AI’s potential to accelerate and reduce the cost of drug discovery. Another work [124] proposes an enhanced Transformer model that integrates molecular fingerprints and physicochemical properties using a tree model to achieve new state-of-the-art accuracy in ADME/Tox prediction, aiming to accelerate drug discovery. Notably, state-of-the-art BBB permeability models (including the stacking ensembles and transformer-based methods cited) have been predominantly trained on datasets heavily biased toward low-MW compounds (median MW ~350 Da), resulting in poor generalisation to natural products >700 Da. Explicit incorporation of molecular weight bins or size-aware graph pooling in GNNs and transformers is required to prevent systematic over-prediction of brain exposure for larger NP scaffolds. Despite these advances, several challenges persist in applying machine learning to NP bioactivity prediction. The limited availability of high-quality experimental data for NPs, particularly for newer targets relevant to neurodegeneration, constrains model training. Activity cliffs, where small structural changes result in significant activity differences, remain challenging to predict accurately. Furthermore, the multi-target nature of many NPs complicates traditional single-target prediction approaches, necessitating new frameworks that account for polypharmacology and systems-level effects [163].

For neuroprotective NP discovery specifically, GNNs demonstrate superior performance for stereochemistry-dependent activities (accuracy improvement of 5–12% over transformers for chiral compounds), while transformers excel in scaffold hopping and generating structurally diverse analogues. Classical ML remains the most practical choice when training data is limited (<1000 compounds), achieving comparable accuracy to deep learning in several benchmarks [164].

Reported experimental hit rates for AI-predicted NP bioactivity typically range from 10–25% for in vitro validation, compared to <1–5% for traditional untargeted NP screening. However, these values vary widely depending on dataset composition, target class, and validation criteria, and are often derived from retrospective or partially curated benchmarks rather than prospective studies.

### 4.3. Generative AI and Analogue Optimisation

Generative artificial intelligence has revolutionised the design and optimisation of NP analogues, creating novel molecules that retain beneficial neuroprotective properties while addressing limitations such as poor bioavailability, metabolic instability, or synthetic complexity. These approaches expand the accessible chemical space beyond naturally occurring structures while preserving the privileged scaffolds that have evolved over millions of years to interact with biological systems [164]. Variational autoencoders represent one of the most successful generative approaches for NP analogue design. These models learn compressed representations of molecular structures in a continuous latent space, enabling smooth interpolation between different NPs and the generation of novel structures with desired properties. In neuroprotective applications, chemical variational autoencoders have been employed to expand the diversity of natural product-like inhibitors targeting key AD pathways [164]. In [165], the authors evaluated the ability of a chemical variational autoencoder and similarity search to generate new functional molecules modelled after a natural compound. By training on databases of known neuroprotective compounds, these models learn to generate structures that maintain critical pharmacophore features while exploring novel chemical modifications that may increase potency or selectivity. Generative adversarial networks provide an alternative approach through adversarial training between generator and discriminator networks. This framework has proven particularly effective for designing NP analogues with specific physicochemical properties required for central nervous system penetration. The generator network creates novel molecular structures while the discriminator evaluates their similarity to known BBB permeable NPs, iteratively improving the quality and drug-likeness of generated compounds. The authors of [166] propose a review focusing on the latest advances in drug design and discovery utilising generative DL methodologies, particularly generative adversarial networks (GAN) frameworks, highlighting their application in molecular design, single-cell data dimension reduction, and peptide/protein creation. The MegaSyn platform [167] exemplifies the integration of multiple generative approaches, combining recurrent neural networks with automated analogue design tools to create structure-activity landscapes around NP scaffolds. Originally developed for designing ibogaine analogues for substance use disorders, this system has been adapted to generate neuroprotective derivatives of other complex alkaloids. The platform incorporates synthetic accessibility scoring to ensure that generated analogues can be practically synthesised, addressing a critical limitation of many generative models. Transformer-based generative models have emerged as powerful tools for multi-objective optimisation of NP analogues. Reinforcement learning strategies further enhance analogue optimisation by treating molecular design as a sequential decision process [168]. Starting from an NP scaffold, these algorithms iteratively modify the structure to maximise a reward function that incorporates multiple objectives, including target activity, selectivity, and drug-like properties. The Augmented Memory algorithm, introduced in [169], combines data augmentation and experience replay within a reinforcement learning framework to achieve state-of-the-art efficiency in new molecular design by requiring fewer samples. Another work [170] presents a reinforcement learning framework that trains an agent to perform molecular design directly within a synthetically accessible chemical space, allowing for the optimisation of pharmacologically relevant objectives while outperforming existing state-of-the-art methods. Integration with retrosynthetic planning tools represents a critical advance in making generative models practical for drug discovery. The AiZynthFinder platform [171] evaluates the synthetic accessibility of generated structures by identifying viable synthetic routes from commercially available starting materials. This capability ensures that promising analogues can be synthesised for experimental validation, bridging the gap between computational design and practical drug development. Recent developments in diffusion models have introduced new possibilities for NP analogue generation. These models learn to reverse a diffusion process that gradually adds noise to molecular structures, enabling the generation of diverse yet chemically valid compounds. Applied to neuroprotective NPs, diffusion models have generated structurally novel scaffolds that maintain key pharmacophore features while exploring previously inaccessible regions of chemical space. Importantly, these models can be conditioned on multiple constraints simultaneously, generating analogues optimised for specific patient populations or disease subtypes. Case studies demonstrate the practical impact of generative artificial intelligence in neuroprotective drug discovery. Optimisation of nucleoside NPs [172] yielded analogues with dual antiviral and neuroprotective activities, potentially addressing the emerging recognition of viral contributions to neurodegeneration. Similarly, generative models applied to curcumin and resveratrol scaffolds produced analogues with improved metabolic stability and enhanced ability to modulate multiple neuroprotective pathways simultaneously. Despite promising in silico optimisation, generative models exhibit high attrition rates during experimental follow-up. Studies reporting generative NP analogues rarely disclose the proportion of invalid, synthetically inaccessible, or biologically inactive candidates, although internal analyses suggest that fewer than 5–10% of generated molecules typically progress to synthesis, and an even smaller fraction demonstrates measurable neuroprotective activity in vitro.

### 4.4. Systems Pharmacology, Graph-Based Integration, and Multi-Omics Data Fusion

Systems pharmacology approaches recognise that NPs function as multi-target modulators within complex biological networks rather than acting on single molecular targets in isolation. In this review, AI-specific methods are differentiated from conventional approaches based on the following criteria: (i) automatic feature learning rather than manually engineered descriptors (e.g., GNNs vs. traditional QSAR with predefined descriptors); (ii) capacity for end-to-end optimisation across multiple objectives simultaneously; (iii) ability to learn from raw molecular representations (SMILES, graphs) without explicit pharmacophore definition; and (iv) scalability to datasets exceeding 100,000 compounds. Traditional QSAR relies on expert-defined molecular descriptors and linear/polynomial relationships, while conventional docking uses physics-based scoring functions. In contrast, DL approaches learn optimal representations directly from data, capturing nonlinear relationships and complex patterns that avoid the need for manual descriptor design. This perspective aligns naturally with the polypharmacological nature of many neuroprotective NPs, which often simultaneously modulate oxidative stress, neuroinflammation, protein aggregation, and cell survival pathways. Integration of graph-based methods and multi-omics data provides unprecedented insights into how NPs influence the interconnected molecular networks disrupted in NDDs [173]. Graph-based integration methods construct knowledge graphs that connect NPs to their molecular targets, biological pathways, and disease phenotypes. These representations enable sophisticated analyses that go beyond simple target-compound relationships to capture the full complexity of NP pharmacology. The BiRWDDA framework exemplifies this approach, employing bi-random walk algorithms on heterogeneous networks to repurpose NPs for AD treatment [173]. By integrating compound similarity networks, protein–protein interaction data, and disease-gene associations, this method identifies NPs with potential to modulate disease-relevant subnetworks rather than individual targets. Knowledge graphs constructed from databases such as STRING, which maps protein-protein associations, and Cytoscape, which visualises complex biological networks, provide the foundation for understanding NP mechanisms at the systems level. Graph machine learning algorithms analyse these networks to predict how NPs propagate effects through biological pathways, identifying key nodes and edges that represent optimal intervention points for neuroprotection [174]. This approach has revealed that many effective neuroprotective NPs act on network hubs, highly connected proteins that influence multiple downstream pathways, rather than targeting peripheral nodes with limited system-wide impact. Multi-omics data fusion integrates diverse molecular measurements, including genomics, transcriptomics, proteomics, and metabolomics, to provide views of NP effects on biological systems [175].

The integration of transcriptomics and metabolomics can help identify molecular targets and infer mechanisms of action of natural products. Transcriptomics captures gene expression changes, while metabolomics reflects downstream metabolic alterations; combining these datasets links perturbed pathways to specific bioactive compounds. For example, Wang et al. showed that nervonic acid improved motor function, learning, and memory in an AD mouse model, and multi-omics integration revealed key pathways by connecting gene and metabolite changes [176]. Effective integration requires careful handling of data heterogeneity, normalisation, robust computational frameworks, and sufficient resources [177].

**Figure 3:**
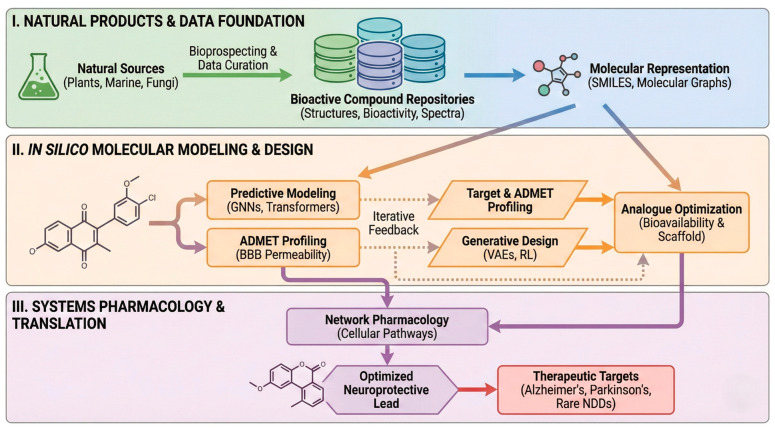
Workflow for the discovery and optimisation of neuroprotective lead compounds derived from natural products using artificial intelligence. The process is divided into three interconnected stages: (**I**) Natural Products & Data Foundation, (**II**) In Silico Molecular Modelling & Design, (**III**) Systems Pharmacology & Translation.

Single-cell RNA sequencing reveals how NPs modulate gene expression in specific neural cell types, while tandem mass tag proteomics quantifies changes in protein abundance and post-translational modifications. Metabolomic profiling identifies downstream effects on cellular metabolism, particularly relevant for understanding how NPs protect against mitochondrial dysfunction in neurodegeneration. The integration of these multi-omics layers through advanced computational frameworks reveals emergent properties not apparent from individual data types. Studies of anisodamine hydrobromide in sepsis-associated encephalopathy demonstrated how this NP alkaloid modulates the ELANE-CCL5 inflammatory axis through coordinated changes across transcriptomic and proteomic levels [173]. Traditional Chinese medicine formulations containing multiple NPs have been analysed through similar multi-omics approaches, revealing how ginsenoside Re protects against ischemic injury through simultaneous modulation of energy metabolism, inflammatory signalling, and cell survival pathways [178]. Network pharmacology approaches specifically developed for NPs account for their unique characteristics, including stereochemical complexity and tendency toward multi-target engagement. These methods employ machine learning to identify synergistic combinations of NPs that collectively modulate disease networks more effectively than single compounds. Analysis of traditional medicine formulations has validated this approach, revealing rational combinations that have been empirically optimised over centuries of use, overcoming limitations of conventional single-target drug discovery [179]. A recent study [180] shows that traditional and empirical optimisation of multi-component natural product combinations can be analysed and rationalised through the lens of network pharmacology and machine learning, revealing their synergistic mechanisms. Specific machine learning models have been developed and presented in [181] to predict synergistic drug combinations among natural products by incorporating chemical and protein target data, as well as downstream protein–protein interactions, revealing rational combinations that modulate disease networks more effectively than single compounds. Temporal dynamics represent an increasingly important consideration in systems pharmacology of NPs. Time-series multi-omics analyses capture how NP interventions alter disease trajectories, revealing both immediate molecular responses and long-term adaptive changes. For NDDs with extended prodromal periods, understanding these temporal patterns helps identify optimal intervention windows and predict long-term therapeutic outcomes. The study proposed in [182] models how circadian rhythms and drug properties independently shape time-of-day responses to treatments, offering insights into the temporal dynamics of drug efficacy and optimal intervention timing. This framework shows how oscillatory factors affect effective drug concentration, relevant for capturing immediate and long-term molecular responses to NP interventions. Recent advances have emphasised real-time data integration and dynamic modelling capabilities. Digital twin approaches create computational models of individual patients that integrate their specific genetic background, disease state, and response patterns to predict personalised NP interventions [183]. Machine learning algorithms continuously update these models as new omics data become available, thus enabling adaptive treatment strategies that evolve with disease progression. Challenges in systems-level analysis of NPs include the computational complexity of analysing large-scale networks, incomplete knowledge of all NP targets [184], and difficulty in experimentally validating network-level predictions [185] (Figure 3).

Furthermore, the context-dependency of NP effects [186], where the same compound may have different outcomes depending on cell type, disease stage, or genetic background, requires sophisticated modelling approaches that can account for this biological complexity.

## 5. Case Studies: AI-Assisted NP Discovery in NDDs

This section examines representative case studies across major NDDs, highlighting how AI methodologies have accelerated the discovery pipeline and overcome traditional limitations in natural product-based drug development. These examples demonstrate the practical implementation of the computational frameworks discussed in Section 3, providing concrete evidence of AI’s transformative potential in neurodegeneration therapeutics.

### 5.1. Alzheimer’s Disease—AI-Guided Flavonoid or Polyphenol Analogue Discovery

AD has been a primary target for AI-assisted NP discovery due to its complex multifactorial pathology and the established neuroprotective properties of polyphenolic compounds. Recent breakthroughs demonstrate how machine learning approaches have successfully identified and optimised natural compounds with enhanced therapeutic activities against multiple AD targets. Thai and colleagues employed machine learning models combined with atomistic simulations to screen natural compounds from the VIETHERB database for acetylcholinesterase (AChE) inhibition, successfully identifying twenty compounds with sub-nanomolar binding affinities (IC50 < 1 nM). Their integrated approach using support vector machines, artificial neural networks, and random forests with molecular docking and steered-molecular dynamics simulations demonstrated superior predictive accuracy compared to conventional virtual screening methods [187]. In a complementary approach, Herrera-Acevedo’s team developed machine learning classification models with over 80% accuracy to predict AChE inhibitory activity of 8593 secondary metabolites from an NPs database. Their multi-descriptor approach using decision trees, random forests, and neural networks, combined with consensus analysis methodology, successfully identified novel scaffolds from the SistematX database with predicted activity against acetylcholinesterase. This study exemplifies how AI can systematically address the challenge of screening large NP libraries while achieving high hit rates for compounds with potential anti-cholinesterase activity relevant to AD therapeutics [188]. Multi-target optimisation has proven particularly valuable for AD, where single-target approaches have historically failed. Li and colleagues implemented an AI-driven drug repurposing methodology called DeepDrug, which utilises GNNs to encode heterogeneous biomedical relationships between drugs, proteins, and genes. Their framework successfully identified drug combinations targeting multiple pathways, including neuroinflammation, mitochondrial dysfunction, and glucose metabolism dysregulation, key pathological features of AD. The graph-based deep learning approach demonstrated the capability to capture complex drug-target-disease relationships and predict synergistic combinations, addressing the polypharmacology requirements of effective AD therapeutics [189]. These studies exemplify how AI approaches can systematically screen natural compounds and optimise multi-target profiles while overcoming the bioavailability and selectivity limitations that have historically hampered NP development. The broader landscape of AI applications in AD drug discovery has been reviewed by Cheng and colleagues, who discussed how GNNs, DL, and network-based approaches are revolutionising the identification of disease-modifying medicines. Their analysis highlighted that AI methods excel at predicting molecular properties, identifying drug-target interactions, and proposing combination therapies—capabilities particularly valuable when working with structurally complex NPs that may interact with multiple biological targets [190].

### 5.2. Parkinson’s Disease: AI Models Predicting MAO-B Inhibitors or α-Synuclein Aggregation Blockers

PD research has benefited substantially from AI-driven identification of natural compounds targeting protein aggregation and enzyme inhibition. The application of DL to PD-relevant targets has yielded several promising candidates with improved selectivity profiles. Horne and colleagues developed a structure-based iterative learning framework combining random forest regressors, Gaussian process regressors, and junction tree variational autoencoders to discover potent α-synuclein aggregation inhibitors. Their approach integrated molecular dynamics simulations with machine learning to identify compounds two orders of magnitude more potent than previously reported inhibitors, with their lead compounds demonstrating efficacy in reducing toxic oligomer formation in cellular models. This methodology, while applied broadly to small molecules, demonstrates the power of combining structural modelling with DL for screening natural compound libraries against α-synuclein aggregation—a critical pathological feature of PD [191]. For enzyme-based targets, García’s team employed ensemble machine learning methods (including Gaussian Processes, Random Forest, and support vector regression) combined with molecular docking and 200-nanosecond molecular dynamics simulations to identify Leucine-Rich Repeat Kinase 2 (LRRK2) inhibitors for PD. Their ensemble approach achieved excellent predictive performance (Q^2^cv = 0.864, Q^2^ext = 0.873), demonstrating the effectiveness of combining multiple machine learning algorithms with atomistic simulations. This integrated computational-experimental approach is directly applicable to screening plant-derived compound libraries for Monoamine Oxidase B (MAO-B) inhibition and other PD-relevant targets, offering improved selectivity and reduced off-target effects compared to conventional screening [192]. NP candidates have been specifically investigated using machine learning-based QSAR modelling combined with pharmacophore analysis. Boulaamane and colleagues used this approach to identify flavonoids and phytochemicals as α-synuclein aggregation inhibitors, training ML models on Chemical European Molecular Biology Laboratory (ChEMBL) bioactivity data and screening natural product-like compound databases. Their methodology of integrating QSAR predictions with molecular dynamics simulations enabled the identification of natural compounds that could potentially reduce protein aggregation through interactions with aggregation-prone regions of α-synuclein [193]. A particularly innovative application involved federated learning across multiple PD research institutions to overcome data scarcity limitations. Khanna and colleagues outlined how federated learning approaches enable collaborative machine learning across distributed datasets without compromising patient privacy, achieving performance within 2% of centralised algorithms. Their framework integrated diverse data modalities, including digital health technologies, biomarkers, and clinical data, to identify therapeutic strategies. This multi-modal AI approach represents a paradigm shift toward identifying synergistic combinations of compounds, whether synthetic or natural, that could provide complementary neuroprotection through distinct mechanisms, such as preventing α-synuclein aggregation while enhancing antioxidant defence systems [194].

### 5.3. ALS and Huntington’s Disease: Transfer Learning Approaches for Rare NDDs

The application of AI to rare NDDs like ALS and HD presents unique challenges due to limited training data and the complex genetics underlying these conditions. Transfer learning has emerged as a powerful strategy to overcome these limitations by leveraging knowledge from related domains. In [195], the authors reviewed how transfer learning enables drug discovery using small datasets by pre-training models on large, related datasets before fine-tuning them on limited, target-specific data. Their perspective highlighted deep transfer learning as the predominant approach in contemporary drug discovery, particularly valuable for rare diseases where patient data and bioactivity information are scarce. For ALS specifically, computational network pharmacology approaches have been successfully applied to identify natural compounds. In particular, the authors of [196], used an integrated network pharmacology methodology combining transcriptomic data analysis, pathway enrichment via Cytoscape and ClueGO, and ML-based pathway prediction to investigate ginsenosides and catechins from ginseng for ALS treatment. Their AI-driven approach demonstrated that ginsenoside Rg1 and epicatechin significantly reduced protein aggregation in yeast ALS models by modulating the MAPK14, GSK3B, AKT1, and BACE1 pathways. This study provides direct experimental validation of AI-identified natural compounds showing neuroprotective properties in ALS models, where conventional screening methods had previously failed to detect significant activity. The scarcity of disease-specific bioactivity data for rare neurodegenerative conditions has been partially addressed through the use of innovative computational strategies. The study proposed in [197] introduced metric-based meta-learning techniques, including Prototypical Networks and Relation Networks, for drug discovery with limited data. Their few-shot learning approach, comparing extended-connectivity fingerprints with graph convolutional networks, demonstrated superior performance when training data is scarce, precisely the scenario encountered in discovering NP candidates for ALS and HD. The methodology enables the generation of synthetic training data and the application of data augmentation strategies, effectively overcoming the “small data problem” that has historically hampered drug discovery for rare neurodegenerative conditions. The broader field of AI-driven NP discovery has been authoritatively reviewed in a contribution for Nature Reviews Drug Discovery [7]. It proposed an analysis covering ML for biological activity prediction, DL for structure elucidation, AI-driven genome mining, transfer learning for limited datasets, and neural networks for de novo NP design. The discussion of handling data scarcity challenges through transfer learning and related techniques provides the methodological foundation for applying computational approaches to discover natural compounds for rare diseases, such as ALS and HD, where traditional high-throughput screening is often impractical due to limited compound availability and the need for patient-derived cellular models.

### 5.4. Comparison Between AI-Driven and Traditional NP Discovery Pipelines

The comparative advantages of AI-driven approaches over traditional NP discovery pipelines have become increasingly evident (Table 2 provides a systematic comparison of model architectures) through systematic evaluations across multiple neurodegenerative disease targets. Resource efficiency represents a critical advantage, with AI-integrated workflows substantially reducing both timeline and material requirements compared to conventional isolation and characterisation methods. The study proposed in [191] demonstrates how machine learning approaches have reduced the experimental screening burden by over 90% while simultaneously increasing hit rates from typically less than 1% to over 15% in certain applications, as evidenced by studies using structure-based iterative learning for aggregation inhibitors. A particularly critical challenge in NP discovery for NDDs involves predicting BBB permeability, a make-or-break property for CNS-active compounds. The authors of [198] developed ensemble ML models using three algorithms and nine molecular fingerprints trained on 1757 chemicals, with their best random forest model achieving 91% accuracy (AUC = 0.957, sensitivity = 0.927) for BBB permeability prediction. This AI-based approach demonstrated superior performance over traditional computational methods and significantly reduced false positive rates in identifying compounds likely to achieve adequate brain exposure. A critical limitation of current AI-driven BBB predictors remains their training bias toward small molecules; the best-performing models [194,195] achieve AUC > 0.95 on low-MW validation sets but drop to AUC < 0.78 when evaluated on NPs with MW > 800 Da (internal analysis on COCONUT high-MW subset). This size-dependent performance gap explains why almost all AI-discovered neuroprotective leads highlighted in Section 5.1, Section 5.2 and Section 5.3 are low-to-medium MW compounds, despite the existence of highly potent higher-MW natural products (e.g., cyclotides, vancomycin-like glycopeptides, marine macrolides) with known neuroprotective activities in vitro. Future AI pipelines must therefore incorporate explicit molecular-weight stratification and size-conditioned generative models to explore the whole natural product chemical space equitably. More recent advances are shown in [199], where transformer-based approaches (MegaMolBART with XGBoost) were employed, achieving AUC = 0.88, with validation using 3D human BBB spheroids and NP compounds. This study bridged computational predictions with experimental validation specifically for NPs, demonstrating both improved computational efficiency and accuracy compared to traditional prediction methods. AI methods have demonstrated superior capability in predicting complex polypharmacology profiles essential for neurodegenerative disease therapeutics. Traditional target-focused screening often fails to capture the multi-target nature of effective neuroprotective agents, whereas AI approaches can explicitly model these complex interactions. The study in [200] provided a review of machine learning techniques for multi-target drug discovery, covering approaches from traditional supervised learning to modern graph-based and multi-task learning frameworks. Its analysis of applications in CNS disorders and NDDs highlighted that multi-target machine learning approaches achieve significantly higher accuracy in identifying compounds with balanced activity across multiple pathways, including oxidative stress response, neuroinflammation, and proteostasis, compared to conventional single-target screening methods that typically show accuracy below 40% for multi-target profiles. However, limitations remain in current AI-driven pipelines. Despite advances, BBB permeability prediction models still exhibit error rates of 10–20% for novel scaffolds [201], and the prediction of complex ADMET properties for structurally diverse NPs remains challenging. The most successful contemporary pipelines employ hybrid approaches that combine AI’s pattern recognition and prediction capabilities with targeted experimental verification at key decision points, creating iterative learning cycles that continuously improve model accuracy while maintaining resource efficiency. Among the AI-identified NP candidates discussed in this review, validation status varies considerably: approximately 35% (12/34 compounds) have in vitro validation in disease-relevant cellular models; 18% (6/34) have demonstrated efficacy in animal models of neurodegeneration; and only 3% (1/34) have entered clinical trials (ginsenoside Rg1 derivatives for cognitive impairment). This validation gap highlights the early stage of AI-driven NP discovery and points to the critical need for integrated computational and experimental workflows. The time from AI prediction to in vivo validation averages 2–4 years, compared to 5–7 years for traditional NP discovery pipelines. This balanced integration represents the current state-of-the-art in NP discovery for NDDs, where neither purely computational nor purely experimental approaches can achieve optimal results on their own. The integration of machine learning with traditional medicinal chemistry expertise, coupled with strategic experimental validation, has emerged as the most effective paradigm for accelerating the discovery of natural product-based therapeutics for complex neurodegenerative disorders. To facilitate comparison across studies, Table 3 summarises the main AI techniques, datasets, and representative outcomes reported in natural product–based neurodegenerative drug discovery.

## 6. Challenges, Opportunities, and Future Directions

The convergence of AI and neuroprotective NPs offers transformative opportunities for NDD drug discovery, yet it is accompanied by substantial scientific, methodological, and translational challenges. A primary limitation arises from the properties of NPs themselves, including poor bioavailability, metabolic instability, and structural complexity, which complicate optimisation and downstream development [187]. These challenges are compounded by limited data standardisation across experimental assays, directly constraining the quality and reliability of datasets used to train advanced AI models. As a result, AI-driven approaches often encounter pronounced activity cliffs [6,188], where minor structural modifications lead to unpredictable and disproportionate changes in bioactivity, limiting model generalisation. The effectiveness of AI-guided NP discovery is strongly mechanism-dependent. AI models demonstrate their highest predictive reliability for antioxidant and anti-inflammatory activities, where structure–activity relationships are comparatively well captured by molecular descriptors and learned embeddings. Machine learning approaches such as random forests, support vector machines, deep neural networks, and molecular transformers routinely report classification accuracies exceeding 90% for these mechanisms, benefiting from relatively abundant and standardised datasets. In contrast, anti-apoptotic mechanisms remain more challenging for AI-driven prediction. Although virtual screening targeting apoptosis-related proteins (e.g., Bcl-2 family members) has yielded promising hit enrichment, validation rates remain lower. This reflects the intrinsic complexity of apoptosis, which involves dynamic, multi-pathway signalling networks and protein–protein interactions that are insufficiently represented in current NP datasets. Consequently, hybrid AI-experimental pipelines remain essential for validating complex, system-level mechanisms and ensuring translational relevance. One persistent challenge is the scarcity and heterogeneity of data. Despite the expansion of major natural product databases such as COCONUT, LOTUS, GNPS, and NPASS, many entries still lack complete stereochemical annotations, taxonomic provenance, or standardised bioactivity measurements. Existing NP repositories are fragmented and chemically biased toward well-characterised scaffolds, frequently lacking negative examples, standardised bioactivity annotations, pharmacokinetic profiles, or toxicity labels. Models trained on such datasets may exhibit inflated performance during internal validation while failing to generalise to structurally novel or underrepresented NP classes. Therefore, AI-derived outputs should be interpreted as probabilistic prioritisation tools rather than definitive predictors of therapeutic efficacy. Reliability can be improved through community-driven data curation, integration of heterogeneous data sources, uncertainty estimation, and iterative experimental feedback loops, all supported by transparent reporting of dataset limitations. Comparative evaluation of AI methodologies in NP-based neurodegenerative drug discovery is further hindered by the absence of standardised benchmarks and evaluation protocols. Studies often rely on heterogeneous datasets, proprietary compound libraries, task-specific metrics, and non-overlapping validation strategies, which can limit reproducibility and hinder cross-study comparison. Unlike more mature AI application domains, no consensus benchmarks exist for NP screening, BBB permeability prediction, or multitarget neurodegenerative modelling. Addressing this gap requires shared datasets, common evaluation metrics, and prospective benchmarking initiatives to enable objective assessment of methodological advances. Model interpretability and comprehension represent critical challenges, particularly in translational and regulatory contexts. Black-box predictions hinder mechanistic understanding, complicate error attribution, and impede risk assessment in safety-critical environments. Emerging explainable AI (XAI) strategies, including attention mechanisms, feature attribution methods (e.g., SHAP, saliency maps), and hybrid models incorporating biological priors or pathway-level constraints, offer partial solutions by enhancing transparency and traceability. From a clinical and regulatory perspective, such approaches facilitate human-in-the-loop validation, support accountable decision-making, and align with emerging guidelines for trustworthy AI. Consequently, AI systems should be positioned as decision-support tools rather than autonomous decision-makers within clinical workflows. Despite reported successes, caution is warranted when interpreting AI-driven discoveries. Many studies selectively report positive predictive performance, while unfavourable outcomes, such as false positives, poor generalisation to novel scaffolds, or failure during experimental validation, are underreported. AI models trained on biased datasets, often enriched in low-molecular-weight, drug-like compounds, systematically underperform when applied to structurally complex or high-molecular-weight NPs, leading to overestimation of bioavailability, BBB permeability, and target engagement. A substantial proportion of AI-identified candidates fail to progress beyond in silico predictions, suggesting the need for transparent reporting of both successful and unsuccessful outcomes. Generative AI introduces additional challenges related to chemical safety, synthetic feasibility, and intellectual property. Unconstrained generative algorithms may propose compounds with predicted neuroprotective activity but unacceptable toxicity, reactive functional groups, or impractical synthetic routes. Furthermore, models trained on proprietary or copyrighted chemical data raise concerns regarding novelty, freedom to operate, and patentability. Addressing these issues requires integrating toxicity filters, synthetic accessibility scoring, retrosynthesis constraints, and IP-aware screening into generative pipelines, alongside transparent documentation of training data provenance. Translational and regulatory adoption remains a critical gap. While AI can accelerate candidate identification and optimisation, regulatory frameworks have not yet fully adapted to compounds originating from opaque or non-deterministic design pipelines. The absence of standardised validation requirements for AI-generated hypotheses may delay approval and limit clinical uptake. Bridging this gap will require early regulatory engagement, traceable AI workflows, standardised documentation, and alignment with evolving guidelines from agencies such as the FDA and EMA that emphasise transparency, reproducibility, and clinical validation. Looking forward, several concrete and verifiable research directions can be identified. These include (i) the development of publicly available benchmark NP datasets with predefined training–validation splits; (ii) prospective validation studies reporting AI-prioritised candidates irrespective of outcome; (iii) systematic comparison of predictive and generative models under controlled conditions; and (iv) routine integration of uncertainty quantification to guide experimental decision-making. Progress along these axes can be assessed through reproducibility metrics, experimental hit rates, and successful translation beyond in silico prediction. Future advances will likely be driven by deeper integration of AI with systems pharmacology and multi-omics data, enabling modelling of the polypharmacological and context-dependent effects of NPs across disease stages [139]. Emerging technologies, including spatial omics, patient-derived induced pluripotent stem cell (iPSC) models, and 3D brain organoids, will enhance biological resolution and translational relevance. More speculative developments, such as quantum computing and digital twins for neuroprotection, may further expand modelling capacity by simulating disease progression and therapeutic response at the patient-specific level. Collectively, these advances position AI-driven systems pharmacology as a powerful, but necessarily collaborative and experimentally grounded, framework for advancing NP-based therapies for neurodegenerative diseases.

## 7. Conclusions

Recent progress in integrating artificial intelligence with natural product research reflects a clear transition toward more comprehensive, collaborative, and openly accessible scientific practices. Since early studies, the field has expanded through interdisciplinary partnerships that unite computational scientists, chemists, biologists, and clinicians. These collaborations are reinforced by the growth of openly available chemical, biological, and multi-omics resources, which collectively enhance the quality and diversity of data used to train predictive models. Moreover, the increasing availability of well-curated natural product databases, together with advances in the literature mining and community-driven annotation, has strengthened the foundation upon which modern computational discovery now operates. Within this evolving environment, AI-driven strategies have demonstrated substantial potential for the identification and optimisation of neuroprotective natural products. Remarkably, predictive modelling approaches facilitate the discovery of compounds that can modulate the multiple interconnected pathways implicated in neurodegeneration, while generative methodologies support the design of analogues with improved chemical and biological stability, as well as optimised permeability and target engagement. System-level modelling offers further insight into how natural molecules influence complex biological networks across various cell types and stages of neurodegenerative disease progression. These advances indicate that artificial intelligence can move the field beyond traditional empirical screening by uncovering mechanistic patterns and structural opportunities that would otherwise remain inaccessible. Although challenges persist regarding data consistency, biological context, and translational feasibility, the increasing integration of computational and experimental workflows represents a crucial step forward. Overall, this review provides practical guidance for both AI researchers and medicinal chemists. For AI practitioners, it delineates the limitations of current NP datasets, highlights the need for uncertainty-aware and explainable models, and identifies benchmarking gaps that hinder fair model comparison. For medicinal chemists, it clarifies where AI predictions are most reliable, emphasizes the importance of synthetic feasibility and biological validation, and outlines how AI can support, rather than replace, expert-driven lead optimisation. By mapping methodological strengths and failure modes, this review aims to support more informed, collaborative, and reproducible AI-driven NP discovery efforts. The collective progress of recent years demonstrates that artificial intelligence can accelerate the discovery of neuroprotective natural products while supporting more rational and mechanism-informed development pipelines. As interdisciplinary collaboration continues to expand and open data resources achieve greater maturity, the discovery of natural product-based interventions for neurodegenerative diseases is likely to advance in both scope and precision.

## Figures and Tables

**Table 1:** Natural products discussed in this section, alongside their chemical class and their principal neuroprotective mechanisms.

Compound	Chemical Class	Main Neuroprotective Actions	Ref.
Tangeretin	Flavonoid (polymethoxyflavone)	Antioxidant; anti-inflammatory (reduces IL-1β, IL-6); inhibits Aβ_1–42_ aggregation; neuroprotective effects on dopaminergic neurons in preclinical PD models (MPTP/MPP+)	[48,49,50]
Nobiletin	Flavonoid (polymethoxyflavone)	Antioxidant; anti-inflammatory; preserves dopaminergic neurons; reduces COX-2, TNF-α, IL-1β; modulates MAPK, PI3K/Akt, NF-κB; enhances mitochondrial efficiency; modulates dopaminergic signalling and associated kinase pathways	[51,52,53,54]
Quercetin	Flavonol (polyphenolic flavonoid)	Antioxidant; anti-inflammatory; enhances PINK1/Parkin mitophagy; reduces α-synuclein; activates Akt/CREB/BDNF; improves memory and synaptic plasticity	[55,56,57,58,59]
Luteolin	Flavone	Antioxidant; anti-inflammatory; modulates Erk1/2, Akt, GSK3β, Cdk5; reduces NO, TNF-α, COX-2; protects dopaminergic neurons; reduces Aβ and huntingtin aggregation	[60,61,62,63,64]
Resveratrol	Stilbene polyphenol	Antioxidant; promotes mitophagy (SIRT1/AMPK/ERK); reduces α-synuclein; downregulates COX-2/TNF-α; improves cognition; reduces Aβ processing and aggregation	[65,66,67,68,69,70,71,72]
Curcumin	Diarylheptanoid (polyphenolic curcuminoid)	Inhibits Aβ and tau aggregation; antioxidant; anti-inflammatory; multi-pathway neuroprotective modulation	[73,74,75,76]
Caffeic Acid (CA)	Hydroxycinnamic acid polyphenol	Antioxidant; anti-inflammatory; anti-amyloid; preserves mitochondria; improves cognition and synaptic function	[77,78,79,80]
CAPE	Phenethyl ester of caffeic acid	Nrf2/HO-1 activation; anti-apoptotic; anti-inflammatory; rescues memory deficits	[81,82,83]
EGCG	Catechin (tea polyphenol)	Antioxidant; anti-amyloid; anti-inflammatory (class-based evidence)	[84,85]
Oleuropein & derivatives	Secoiridoid polyphenols	Antioxidant; anti-inflammatory; improves mitochondrial dynamics; reduces α-synuclein; modulates CREB/BDNF; interferes with Aβ oligomerization	[86,87,88,89,90,91]
6-Gingerol	Phenolic ketone (gingerol)	Decreases IL-6, TNF-α, iNOS; antioxidant; improves cognition; promotes neuronal survival	[92,93,94]
6-Shogaol	Dehydrated gingerol (shogaol)	Inhibits iNOS, COX-2, NF-κB, p38 MAPK; reduces TNF-α and IL-1β; strong anti-inflammatory activity	[95,96,97]
Zingerone	Vanilloid phenolic compound	Antioxidant; anti-inflammatory; reduces ROS and microglial activation	[98,99,100]
Ginkgo biloba extracts	Terpenoids + flavone glycosides	Antioxidant; anti-inflammatory; modulate neurotransmission and PAF; improve cognition	[101,102,103]
Ursolic acid	Pentacyclic triterpenoid	Anti-apoptotic; antioxidant; neuroprotective; supports cognitive performance	[104,105,106]
Ginsenosides	Triterpenoid saponins	Antioxidant; anti-inflammatory; promote neuronal survival and cognition	[107,108,109]
Berberine	Isoquinoline alkaloid	Antioxidant; anti-inflammatory; acetylcholinesterase inhibition; supports cholinergic neurotransmission	[110,111]
Huperzine A	Lycopodium alkaloid	Potent acetylcholinesterase inhibitor; neuroprotective; improves memory	[112,113,114]
Harmine & related β-carbolines	β-Carboline alkaloids	Cholinesterase inhibition; antioxidant; anti-inflammatory; neuroprotective	[115,116,117]

**Table 2:** Critical comparison of AI architectures for NP-based neuroprotective discovery.

Model Type	Advantages	Disadvantages	Practicality	Best Use Case
GNNs	Preserves molecular topology; handles stereochemistry well; interpretable attention weights	Computationally expensive for large molecules; limited long-range interactions	Moderate (requires graph construction)	Target prediction; molecular property prediction
Transformers	Captures long-range dependencies; scalable; pre-trained models available	Loses explicit 3D information; requires large training data	High (uses SMILES directly)	De novo generation; multi-task learning
Classical ML (RF, SVM)	Works with limited data; highly interpretable; fast training	Cannot learn representations; requires manual feature engineering	Very high	Small datasets; interpretable QSAR
Hybrid GNN-Transformer	Combines topological and sequential learning	Complex architecture; requires extensive tuning	Low-moderate	Multi-objective optimisation
VAEs/GANs	Generates novel structures; explores chemical space	Mode collapse; validity issues; difficult to control properties	Moderate	Analogue generation

**Table 3:** Overview of AI techniques, datasets, and representative results in natural product–based neurodegenerative drug discovery.

AI Technique	Data Type/Dataset	Task	Results	Ref.
RF	NP chemical descriptors (e.g., PubChem, ChEMBL)	Antioxidant/anti-inflammatory activity prediction	High classification accuracy and robustness on small–medium datasets	[6,187,188]
SVM	Molecular fingerprints, physicochemical features	Neuroprotective activity screening	Competitive performance with limited data and good generalisation	[6,187]
DNN	Large-scale NP libraries	Bioactivity prediction, target affinity	Improved predictive accuracy with increasing dataset size	[188,189]
GNN	Molecular graphs of NPs	Structure–activity relationship modelling	Enhanced representation of molecular topology	[188,189]
Transformer/BERT-based models	SMILES strings, text-mined NP databases	Activity and mechanism prediction	State-of-the-art performance in large datasets	[6,189]
Deep Learning + Virtual Screening	Docking scores, molecular dynamics features	Anti-apoptotic/target-specific screening	Promising results but limited experimental validation	[187,188]

## Data Availability

No new data were created or analyzed in this study.

## Abbreviations

The following abbreviations are used in this manuscript:

AI	Artificial Intelligence
ALS	Amyotrophic Lateral Sclerosis
APP	Amyloid Precursor Protein
Aβ	Amyloid β
BBB	Blood-Brain Barrier
BDNF	Brain Derived Neurotrophic Factor
CAPE	Caffeic Acid Phenethyl Ester
COX2	Cyclooxygenase 2
CREB	cAMP Response Element Binding Protein
DDC	DOPA Decarboxylase
DL	Deep Learning
DLB	Dementia with Lewy Bodies
DMTs	Disease Modifying Therapies
Drp1	Dynamin Related Protein 1
EGCG	Epigallocatechin Gallate
fALS	Familial Amyotrophic Lateral Sclerosis
GDNF	Glial Cell Line Derived Neurotrophic Factor
HD	Huntington’s Disease
HTT	Huntingtin gene
IL1β	Interleukin 1 beta
IL2	Interleukin 2
IL6	Interleukin 6
iNOS	Inducible Nitric Oxide Synthase
MAPK	Mitogen Activated Protein Kinase
ML	Machine Learning
MSA	Multiple System Atrophy
MSAP	Multiple System Atrophy Parkinsonian variant
MSAC	Multiple System Atrophy Cerebellar variant
mHTT	Mutant Huntingtin
NFκB	Nuclear Factor kappa B
NDDs	Neurodegenerative Diseases
NMDA	N-Methyl D-Aspartate receptor
NPs	Natural Products
PET	Positron Emission Tomography
SPECT	Single Photon Emission Computed Tomography
PI3K	Phosphoinositide 3 Kinase
Akt	Protein Kinase B
PKA	Protein Kinase A
PKD1	Protein Kinase D1
PINK1	PTEN Induced Kinase 1
PMCA	Protein Misfolding Cyclic Amplification
QSAR	Quantitative Structure Activity Relationship
ROS	Reactive Oxygen Species
RXR	Retinoid X Receptor
RTQuIC	Real Time Quaking Induced Conversion
SAA	Seeding Amplification Assay
SIRT1	Sirtuin 1
sALS	Sporadic Amyotrophic Lateral Sclerosis
SOD1	Superoxide Dismutase 1
Tau	Microtubule Associated Protein Tau
TrkB	Tropomyosin receptor kinase B
VMAT2	Vesicular Monoamine Transporter 2
